# Characterisation data for open-air processed common water reed (*Phragmites australis*) ash and papyrus (*Cyperus papyrus*) ash

**DOI:** 10.1016/j.dib.2022.108423

**Published:** 2022-06-26

**Authors:** Martin Aluga, Chewe Kambole

**Affiliations:** a Civil Engineering and Construction Department, Copperbelt University, Kitwe, Zambia; b School of Industrial Engineering, Universidad de Valladolid, Valladolid, Spain

**Keywords:** Cement, Common water reed ash (CWRA), Cyperus papyrus ash (CPA), Scanning electron microscope (SEM), Sustainable development

## Abstract

Currently, there are a lot of discussions on the production of sustainable cement for construction purposes, unlike the conventional ordinary Portland cement (OPC), as its production, transportation, and application contribute to the generation of greenhouse gases, hence, climate change. Consequently, limestone, the primary material used to produce OPC, is non-renewable. Therefore, there is a need to use sustainable materials to make cementitious materials to achieve sustainable construction. This has led to a lot of research focussing on the valorisation of agricultural wastes and less economical, no-food lignocellulosic plants in producing sustainable and environmentally friendly cementitious materials commonly known as Supplementary Cementitious Materials (SCMs). The agrowastes ashes include rice husk ash (RHA), sugarcane bagasse ash (SCBA), and corn cob ash (CCA), among others. In contrast, the lignocellulosic plants’ ashes include common water reed ash (CWRA) and cyperus papyrus ash (CPA). There has been the belief that these pozzolanic materials are homogenous. However, these ashes are highly heterogeneous when they undergo microscopic analysis. Therefore, the current data paper provides Laser Diffraction Spectroscopy (LD) for Particle Size Distribution (PSD), Fourier-transform infrared spectroscopy (FT-IR), X-Ray Fluorescence (XRF), and Scanning Electron Microscope (SEM) data for unprocessed CWRA and CPA in the form of tables, micrographs, and figures for microscopic analysis. This data helps characterise and evaluate CWRA and CPA's potential as pozzolanic materials, especially as road construction materials, and will be beneficial for other scientists to better understand unprocessed CWRA and CPA mineral information development biologically inspired materials for biologically inspired materials sustainable development across many disciplines.

## Specifications Table

**Table:** 

Subject	Engineering
Specific subject area	Materials Characterisation
Type of data	Tables, Figures, and Images
How data were acquired	Particle size distribution, spectroscopic, and microscopic data used to classify lignocellulosic bio-pozzolans for engineering applications are explored.
Data format	Raw, Analysed
Parameters for data collection	Particle size distribution (PSD) data were obtained using laser diffraction (LS) mastersizer 2000.FT-IR spectra were obtained using BRUKER TENSOR 27 in 4500–500 cm^−1^.The chemical compositions of CWRA and CPA were characterised by the X-ray fluorescent (XRF) BRUKER model S8 TIGER XRF spectrometer.The Scanning Electron Microscope (SEM) data, the Hitachi FlexSEM 1000, was used after gold plating.
Data source location	Common Water Reed (*Phragmites australis*) were obtained on the banks of River Kafue in Zambia, while Cyperus papyrus (CP) was obtained from the banks of the River Nile in Uganda. Further details of the locations are provided in Table 3.
Description of data collection	The CWR and CP were sun-dried and burned on a hard surface to avoid contamination by foreign materials. After cooling, the CWRA and CPA were sampled in airtight polythene bags for microscopic analysis using Laser Diffraction Spectroscopy (LD) for Particle Size Distribution (PSD), FT-IR, XRF spectroscopy, and SEM.
Data accessibility	The data is available in the article (https://data.mendeley.com/datasets/n3vzfpkt9p/1).

## Value of the Data

• Microscopic analysis requires expensive equipment and is time-consuming. These data fully show the ultra-structures of the unprocessed CWRA and CPA as green pozzolanic materials based on Laser Diffraction, FT-IR, Raman spectra, and SEM micrographs be useful for researchers who do not have access to these types of equipment.
• The data presented here are valuable to researchers investigating the partial replacement of cement in all types of concrete with CWRA and CPA.
• Other researchers may use these data to better understand CWRA and CPA mineral information to develop biologically inspired materials and extract green nanoparticles/nanomaterials.
• The data are relevant for government agencies seeking a classification system to characterise ashes from CWRA and CPA as pozzolanic materials.

## 1 Data Description

The data obtained show particle size distribution (PSD) by Laser Diffraction (LS) method, FT-IR spectra, chemical composition XRF data, and SEM micrographs of CWRA and CPA specimen ashes. The collected data includes three (3) tables, three (3) figures, and raw data to be found on the following link: https://data.mendeley.com/datasets/n3vzfpkt9p/1. The three tables and figures are described within where they appear in the article. The supplementary data No. 1 include the raw data for particle size distribution as obtained from the Mastersizer 2000. The supplementary data No. 2 contains the raw data from the Fourier transform infrared analyses using the BRUKER Tensor 47. The supplementary data No. 3 contains the raw SEM micrographs as obtained from the Hitachi FlexSEM 1000.

### 1.1 Particle Size Distribution

Particle size distribution is a valuable characteristic of materials, especially pozzolanic materials. It defines the reactivity of the material. Coarse materials and fine materials react differently. The most common approach for expressing laser diffraction results is to report the D10, D50, and D90 values based on a volume distribution. Table 1 details the D_10_, D_50_, and D_90_ of CWRA and CPA under different LS conditions, including obscuration. The raw data is provided as Supplementary material No. 1-particle size distribution.

**Table 1:** Details the particle size distribution of CWRA and CPA under different laser diffraction conditions.

No.	Sample Name	d (0.1)	d (0.5)	d (0.9)
1.	CWRA-Unprocessed_1.6bar_60%	4.803	26.759	165.841
2.	CWRA-Unprocessed_1.6bar_60%	6.131	36.793	214.794
3.	CWRA-Unprocessed_1.6bar_60%	6.382	51.440	92.721
4.	CWRA-Unprocessed_1.6bar_60% - Average	5.815	40.707	134.518
5.	Averaged Result_2measurements	5.369	31.449	188.865
6.	CWRA-Unprocessed_1.6bar_50%	4.618	25.575	165.264
7.	CWRA-Unprocessed_1.6bar_50%	4.997	28.424	164.278
8.	CWRA-Unprocessed_1.6bar_50%	10.731	68.923	325.313
9.	CWRA-Unprocessed_1.6bar_50% - Average	5.983	39.417	246.063
10.	Averaged Result_CWRA_unp_1_4_5	4.800	26.900	165.114
11.	CWRA-Unprocessed_1.6bar_40%	4.602	25.115	151.737
12.	CWRA-Unprocessed_1.6bar_40%	5.030	28.261	155.859
13.	CWRA-Unprocessed_1.6bar_40%	4.860	27.572	157.230
14.	CWRA-Unprocessed_1.6bar_40% - Average	4.822	26.949	155.030
15.	CPA-Unprocessed_1.6BAR_40%	5.333	32.124	166.574
16.	CPA-Unprocessed_1.6BAR_40%	6.607	41.848	226.162
17.	CPA-Unprocessed_1.6BAR_40%	6.585	40.597	195.952
18.	CPA-Unprocessed_1.6BAR_40% - Average	6.093	38.015	196.081
19.	CPA-Unprocessed_1.6BAR_50%	5.754	35.013	175.473
20.	CPA-Unprocessed_1.6BAR_50%	6.697	40.614	189.629
21.	CPA-Unprocessed_1.6BAR_50%	6.656	41.108	194.824
22.	CPA-Unprocessed_1.6BAR_50% - Average	6.330	38.854	186.661

### 1.2 FT-IR Spectra

**Fig. 1:**
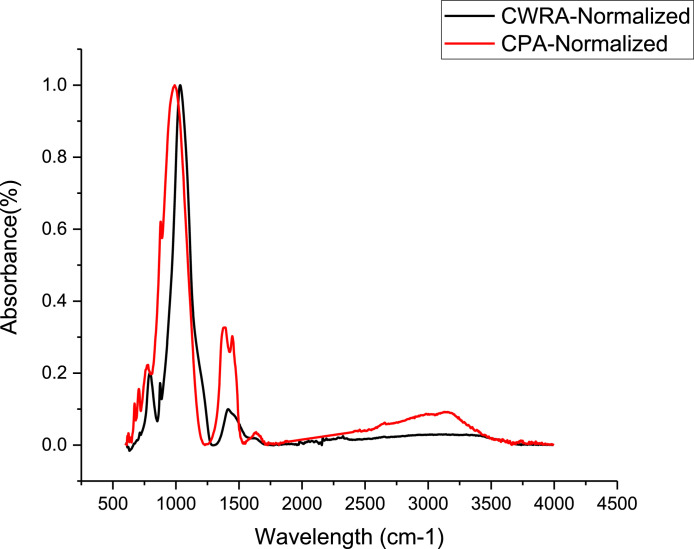
FT-IR Spectra for CWRA and CPA specimen.

FTIR spectral identified the critical chemical compound existing in the unprocessed lignocellulosic bio-pozzolan (CWRA and CPA), as revealed in Fig. 1(https://data.mendeley.com/datasets/n3vzfpkt9p/1) [1,2]. The FT-IR raw data is provided as Supplementary material No. 2-FTIR Raw Data.

### 1.3 X-Ray Fluorescence

The chemical compositions of CWRA and CPA were characterised by the X-ray fluorescent (XRF) BRUKER model S8 TIGER XRF spectrometer. Table 2 shows the results of the experiment which are uploaded on https://data.mendeley.com/datasets/n3vzfpkt9p/1.

**Table 2:** The oxides composition of CWRA and CPA.

Oxide	CPA Concentration	Oxide	CWRA Concentration
SiO_2_	33.70%	SiO_2_	63.40%
K_2_O	28.20%	K_2_O	7.60%
Cl	4.10%	CaO	5.90%
CaO	2.70%	P_2_O_5_	2.60%
P_2_O_5_	2.10%	MgO	2.20%
Na_2_O	1.40%	Cl	0.60%
MgO	1.20%	Al_2_O_3_	0.60%
Al_2_O_3_	0.50%	Fe_2_O_3_	0.40%
MnO	0.30%	SO_3_	0.30%
Fe_2_O_3_	0.30%	MnO	0.10%
SO_3_	0.30%	Na_2_O	0.05%
Br	0.06%	TiO_2_	0.04%
BaO	0.04%	SrO	0.04%
TiO_2_	0.03%	BaO	0.04%
ZnO	0.02%	ZnO	0.01%
SrO	0.02%	ZrO_2_	0.01%
Rb_2_O	0.01%	LOI	16.50%
LOI	24.30%		

### 1.4 Scanning Electron Microscopy

**Fig. 2:**
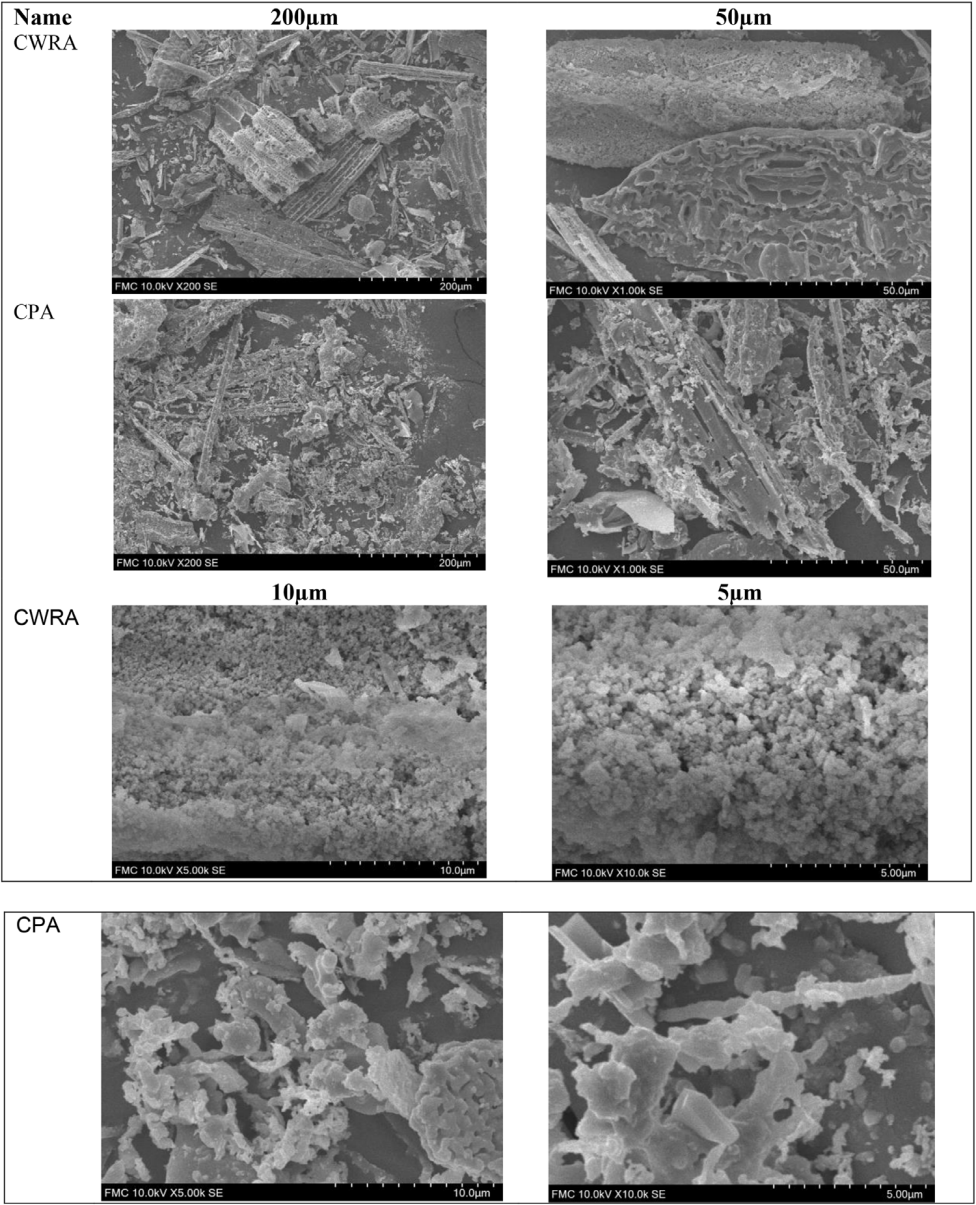
SEM micrographs for CWRA and CPA specimens at different magnifications.

Fig. 2 shows the sampled SEM micrographs for CWRA and CPA specimens. The ImageJ software can analyse the micrographs to determine the diameters, areas and length [3,4]. The raw SEM micrographs are provided as Supplementary material No. 3-SEM Raw Data uploaded at https://data.mendeley.com/datasets/n3vzfpkt9p/1.

## 2 Experimental Design, Materials and Methods

### 2.1 Synthesis of CWRA and CPA

**Fig. 3:**
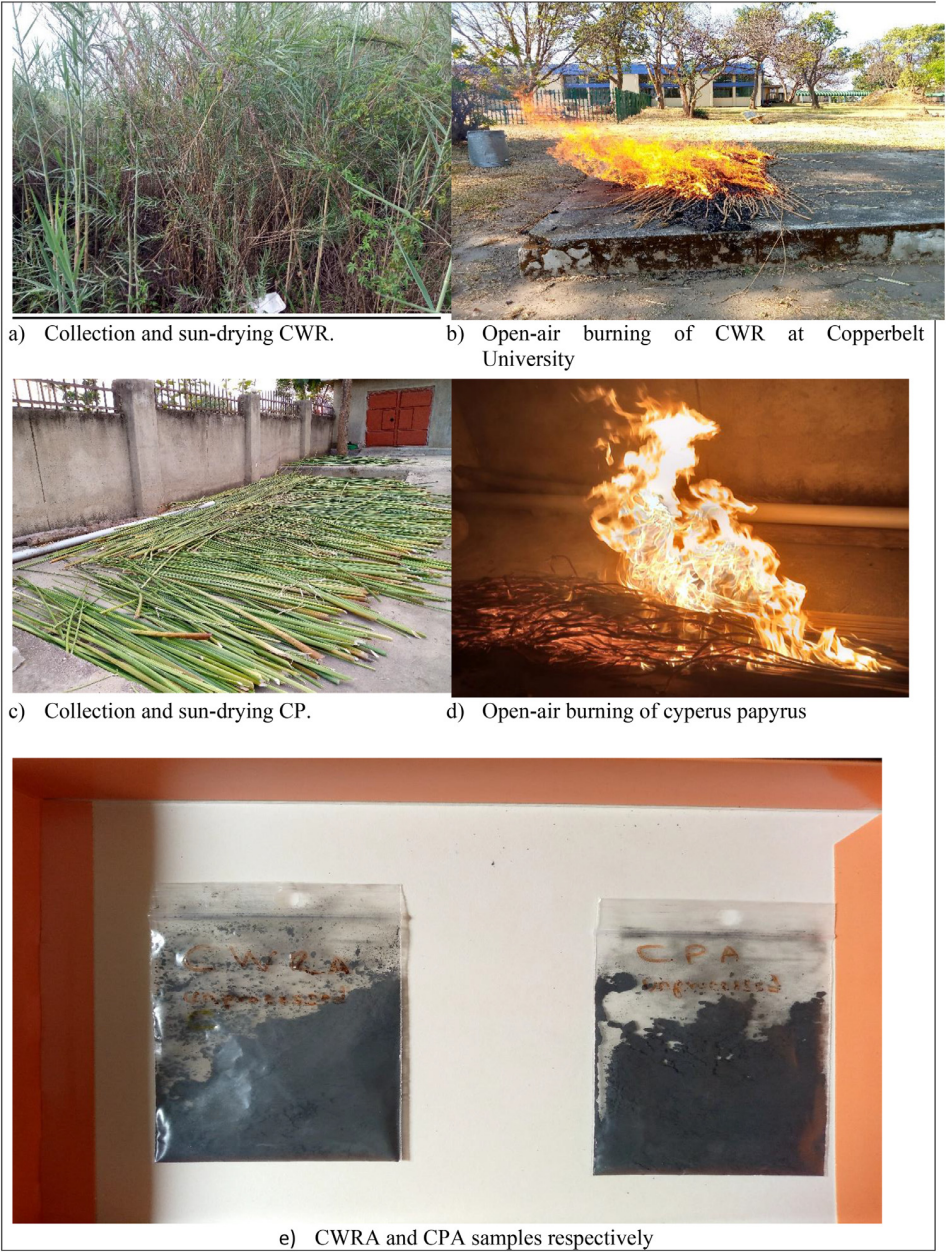
CWRA and CPA specimen preparation (all the photos were taken by author).

The common water reeds were obtained on river Kafue in Zambia, while the cyperus papyrus were obtained from Adjumani on River Nile in Uganda. The details of the locations of collecting theses samples are provided in Table 3. The Fig. 3 shows the process chart for obtaining the open-air processed Common Water Reed (*Phragmites australis*) Ash and Cyperus Papyrus (*Cyperus Papyrus*) Ash. The general process involve cutting the samples from there natural habitats, drying them on hard surface and burning them in open-air under uncontrolled conditions. Therefore, representative samples are collected and packaged in airtight polythene bags for advanced materials caharacterisation.

**Table 3:** Sample collection locations in Uganda and Zambia.

		Coordinates		
Name	Location/Country	Latitude	Longitude	Altitude
Common Water Reeds	Copperbelt, Zambia	12°47′24.36"S	28°15′26.48"E	∼1176m
Cyperus Papyrus	Adjumani, Uganda	3°26′3.61"N	31°39′28.30"E	∼621m

### 2.2 Characterisation of the Unprocessed CWRA and CPA

#### 2.2.1 Laser Diffraction Spectroscopy

Particle size distribution (PSD) data were obtained using laser diffraction (LS) Malvern Mastersizer 2000 as adopted by different authors [5], [6], [7], [8], [9]. The data were obtained at different obscuration between 60% to 40% under constant 1.6 bars.

#### 2.2.2 Fourier-Transform Infrared Spectroscopy

Fourier-transform infrared spectroscopy (FT-IR) spectra were recorded using BRUKER TENSOR 27 in the range of 4500–500 cm^−1^ as reported by different researchers [10], [11], [12]. Several runs were made to get the most uniform spectra.

#### 2.2.3 X-Ray Fluorescence (XRF) Spectroscopy

The chemical compositions of CWRA and CPA were characterised by a high performance X-Ray Flourescent (XRF) spectrometer (Model: S8 TIGER, Bruker, Germany), equipped with an Rh anode X-ray tube (4 kW, 60 kV and 170 mA). A detailed description of this spectrometer is reported by some researchers [13,14].

#### 2.2.4 Scanning Electron Microscope (SEM) Investigation

The Scanning Electron Microscope (SEM) data, the Hitachi FlexSEM 1000, was used after gold plating as used by different studies [15,16].

## Ethical Approval

It is not required for this study as no humans or animals were studied by any of the authors.

## CRediT Author Statement

**Martin Aluga:** Performed conceptualization; Laboratory work and writing – original draft; **Chewe Kambole:** Performed writing – review & editing.

## Declaration of Competing Interest

No competing interests.

## Data Availability

Characterisation Data (PSD, FTIR, XRF and SEM) for Lignocellulosic Biopozzolans (Original data) (Mendeley Data).
